# Internal and external normalization of nascent RNA sequencing run-on experiments

**DOI:** 10.1186/s12859-023-05607-3

**Published:** 2024-01-12

**Authors:** Zachary L. Maas, Robin D. Dowell

**Affiliations:** 1 Department of Computer Science, University of Colorado, Boulder, USA; 2 BioFrontiers Institute, University of Colorado, Boulder, USA; 3 Department of Molecular, Cellular, and Developmental Biology, University of Colorado, Boulder, USA

**Keywords:** Nascent RNA sequencing, Normalization, Bayesian

## Abstract

In experiments with significant perturbations to transcription, nascent RNA sequencing protocols are dependent on external spike-ins for reliable normalization. Unlike in RNA-seq, these spike-ins are not standardized and, in many cases, depend on a run-on reaction that is assumed to have constant efficiency across samples. To assess the validity of this assumption, we analyze a large number of published nascent RNA spike-ins to quantify their variability across existing normalization methods. Furthermore, we develop a new biologically-informed Bayesian model to estimate the error in spike-in based normalization estimates, which we term Virtual Spike-In (VSI). We apply this method both to published external spike-ins as well as using reads at the $3^\prime$ end of long genes, building on prior work from Mahat (Mol Cell 62(1):63–78, 2016. 10.1016/j.molcel.2016.02.025) and Vihervaara (Nat Commun 8(1):255, 2017. 10.1038/s41467-017-00151-0). We find that spike-ins in existing nascent RNA experiments are typically under sequenced, with high variability between samples. Furthermore, we show that these high variability estimates can have significant downstream effects on analysis, complicating biological interpretations of results.

**Supplementary Information:**

The online version contains supplementary material available at 10.1186/s12859-023-05607-3.

## Introduction

Effective normalization is essential for rigorous analysis of high throughput sequencing data. In sequencing data, normalization identifies a set of features that are expected to be invariant between two data sets and leverages these to counteract the effects of systematic experimental bias and technical variation. Broadly, there are only two possibilities for the source of these invariant features: external spike-in controls or an internal invariant set [1, 2]. Whenever possible, external spike-in controls are preferred [3], as they control for more sources of variation by adding a presumably invariant set of data across samples. However, not all data sets contain external spike-ins and they cannot be added *post-facto*. Consequently, a variety of internal normalization methods have been developed [3, 4] which assume some internal feature of the data—typically a set of genes—is invariant between data sets. While most of these techniques were developed for microarrays or RNA-seq, they have been broadly applied to a variety of sequencing assays.

**Fig. 1:**
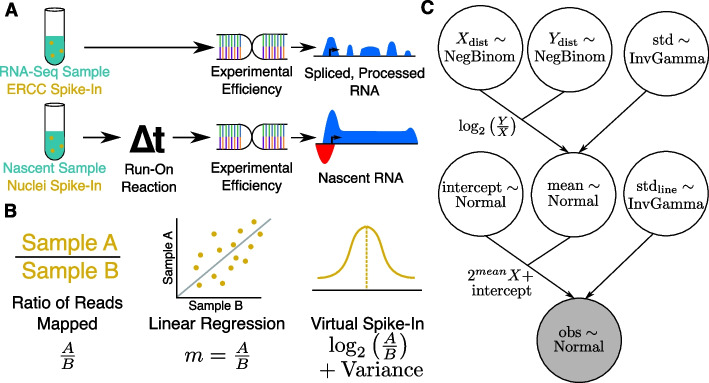
A Bayesian model describing normalization data for nascent RNA sequencing data. **A** Schematic showing typical external control, handling, and resulting data profile differences between RNA-seq (top) and run-on nascent RNA sequencing assays (bottom). Note that run-on efficiency is assumed to be equivalent between spike-in nuclei and experimental nuclei. **B** Quantifying a normalization factor is accomplished either by a naive ratio of total reads approach (left), linear regression (middle), or by the Bayesian model proposed here (right). Linear regression (middle) is more resistant to noise and outliers, but does not provide a reliable way to measure the variance of the normalization estimate. The Bayesian model (right) converts the slope $m = \frac{A}{B}$ to $\log$ space, converting the multiplicative nature of the normalization factor to a linear one, for which normalization factors can be readily inferred as a normal distribution with variance. **C** A plate diagram showing the VSI model as implemented in pymc3. Briefly, we estimate our count distributions *X* and *Y* (top row) with a negative binomial. The ratio of two negative binomial distributions is approximately log-normal, so we derive a normal distribution called *mean* (middle) as the log of the ratio of *Y* and *X* with some variance (top right), estimated as an inverse gamma distributed random variable. With the estimation of the mean established, we then add additional parameters to describe the intercept, and variance of the actual line of best fit. This is done so that the parameter *mean* is estimating an error in log-transformed space, as discussed in Panel (**B**)

One set of protocols in particular—nascent RNA sequencing methods—are prone to large amounts of technical variation [5]. Nascent RNA sequencing protocols, such as global run-on sequencing (GRO-seq) [6], precision run-on sequencing (PRO-seq) [7] and their variations [8, 9], isolate small quantities of recently produced RNAs from actively engaged RNA polymerases [10]. Nascent RNA sequencing samples have a distinct profile relative to RNA-seq (Fig. 1A), resulting from the different phases of the RNA life cycle that they capture. RNA-seq samples from the pool of stable, messenger RNAs (mRNAs) which are predominantly spliced and polyadenylated. These RNAs originate from a relatively small fraction of the genome (exons and UTRs). In contrast, nascent RNA sequencing protocols capture RNA that is still actively engaged with RNA polymerases, meaning the RNAs are pre-splicing and need not be stable. As much of the genome is actively transcribed, nascent transcription protocols recover reads from much larger proportion of the genome (not only exons and introns, but also numerous intergenic regions). Consequently, if both assays are sequenced to the same depth, the equivalent nascent transcription data would have a lower per position depth.

External spike-ins in nascent RNA sequencing are also inherently different than in RNA-seq, leading to more uncertainty in the normalization process (Fig. 1A). The gold standard for spike-ins in RNA-seq is an External RNA Controls Consortium (ERCC) library, which uses a fixed amount of known RNAs which are added to the sample to quantify the variation introduced during sample handling, library preparation and sequencing. Crucially, this RNA spike-in library is introduced in known quantities prior to the experiment. Run-on centric nascent RNA protocols seek to identify the locations of actively engaged RNA polymerases by using marked nucleotides and a run-on reaction. Hence the ERCC spike-ins, by virtue of being mature RNAs, are incompatible with the run-on reaction. Instead, fixed amounts of nuclei from an external organism are typically added to the sample nuclei and then the run-on reaction is employed on the combination of cell types. Thus, the quantity of RNA from the external spike-in is determined by not only the efficiency of the protocol and sequencing, but also the efficiency of the run-on reaction. A necessary but potentially flawed assumption, then, is that all of the run-on reactions have the same efficiency, allowing the reads mapping to the spiked-in nuclei to be treated with the same reliability as an ERCC spike-ins. If an external spike-in is not used, many off-the-shelf RNA-seq tools are used directly for internal normalization [2, 11–13].

Critical to the effectiveness of internal or external normalization are the assumptions about what remains invariant. Notably, when run-on reactions are performed in the presence of a perturbation, nascent RNA sequencing contains a unique internal set of invariant data. RNA Polymerase II loads at the 5$^\prime$ end of a gene and then proceeds through the gene with a relatively consistent processivity [10]. Thus, as first described by Mahat [14], at short time points after a perturbation, changes in transcription are not expected to have reached the 3$^\prime$ end of long genes. Prior work on 3$^\prime$ end normalization applied linear regression to the set of 3$^\prime$ invariant ends and showed this approach was similar to other, presumably invariant, internal gene sets [14, 15]. However, they did not directly compare the approach to external spike-in controls or establish uncertainty bounds on their estimates.

In this work, we set out to compare run-on based 3$^\prime$ normalization to external spike-ins. To this end, we developed a method for quantifying error in the estimation of spike-in normalization. Using this method we compare external spike-ins to internal invariant sets, focusing on the $3^\prime$ subset. We uncovered that most external spike-ins in nascent RNA assays are under-sequenced and potentially unreliable. Additionally, we find that when external spike-ins are of adequate depth and the assumptions of the $3^\prime$ normalization approach are met, the two methods show high correspondence.

## Results

### An algorithm to quantify error in spike-in normalization estimates

When normalizing between samples, there are different approaches to computing normalization factors from the invariant set, whether that set is an external spike-ins or internal [3] (Fig. 1B). The most naive of these is to take the simple ratio of reads mapping to the invariant set between two samples and use that as a normalization factor (Fig. 1B, left). However, this reduces the information contained within the set to a single summary value. The alternative approach is linear regression, where estimates of counts per invariant entity, typically genes, are used as data points for the fitting algorithm and the resulting slope is used as an the normalization factor [3] (Fig. 1B, middle). In this way, transcription levels across different orders of magnitude can be leveraged to give a more accurate normalization factor. Thus, prior work in nascent transcription has often used naive linear regression to estimate normalization factors instead of a simple point estimate [14, 15]. However, to use linear regression, a sample’s spike-ins must be of sufficient depth that a linear relationship exists in the count data. Additionally, naive linear regression does not provide error bounds.

To quantify the error inherent in estimating a normalization factor from data, we developed a hierarchical Bayesian version of the linear regression framework (Fig. 1B, right). Typically linear regression is formulated as:

$$\begin{aligned} y \sim mx+b \end{aligned}$$

which describes the relationship between counts in two samples *x* and *y* in terms of two variables (*m* and *b*) the slope and intercept, respectively. In this framework, the slope (*m*) is interpreted as the best normalization factor between the two samples. In the naive context of normalizing to a spike-in (without considering the error of the estimate), this typically works well, as counts span multiple orders of magnitude and typically form a linear relationship between samples [3]. However, in standard linear regression only a single point estimate for the parameters is obtained.

To quantify the error in the estimated normalization factor, we extend the naive linear model above to incorporate an estimation of the error in log-space, backed by biologically informed count distributions. In the simplest terms, we generate a linear model whose mean is a normal distribution defined by the log-transformed ratio of our read counts [16, 17], plus an intercept term. By using the log-transformed ratio of read counts, we can assume the slope is normally distributed:

$$\begin{aligned} \mu _{\text {slope}} \sim \text {Normal}\left( \text {mean} = \log _{2}{\frac{Y}{X}}, \sigma _{\text {mean}}\right) \end{aligned}$$

Where $\mu _{\text {slope}}$ is the desired mean (normalization factor) and the resulting variance estimate $\sigma _{\text {mean}}$ is then used as an estimate on the error of that normalization factor.

Our model is shown formally as a plate diagram in Fig. 1C. To fully specify the model, we assume the intercept follows a Normal distribution, $\text {Intercept} \sim \text {Normal}$. The input data for this model is formally a counts matrix, *M* where $M_{i,j}$ represents the number of reads in sample *i* in region *j*. For all samples $M_{i}$, we select a single reference sample $M_{r}$ to normalize against. We first model the count data over regions of interest as a Negative Binomial Distribution, as we expect the count distribution to be over-dispersed. This yields two variables—*X* and *Y* which describe to the count distribution of each sample input to the model. Priors for $\sigma$ variables are selected to be uninformative using the conjugate $\text {InvGamma}(1,1)$ [18], while priors for *X* and *Y* are defined as $\mu _X = \text {mean}(M_{i}+1)$ and $\mu _Y = \text {mean}(M_{r}+1)$ to reflect the log-transformed ratio of Laplace smoothed count data.

We call our new method Virtual Spike-In (VSI) and leverage Markov Chain Monte Carlo (MCMC) methods to fit the underlying distributions. The input to the model is a set of data points between two samples, thus this model can also be applied to both external spike-ins and internal invariant sets of regions, such as the unperturbed 3$^\prime$ end of long genes, or to any other set of invariant regions shared between two samples that behave as count data. A technical discussion of implementation details for this model is available in the “Methods” section of this paper.

### Confidence in normalization factor estimates depends on adequate spike-in depth

To assess the correctness of our VSI implementation and approach, we first compare the method to the standard linear regression approach. To this end, we processed samples of human cells with Drosophila spike-ins from a number of previously published studies employing nascent RNA sequencing data [19–32]. After filtering for samples with replicates and a nonzero number of reads mapping to the dm6 Drosophila genome, we were left with $n=180$ samples (Additional file 1: Table S1, see Methods for complete details on data processing).

**Fig. 2:**
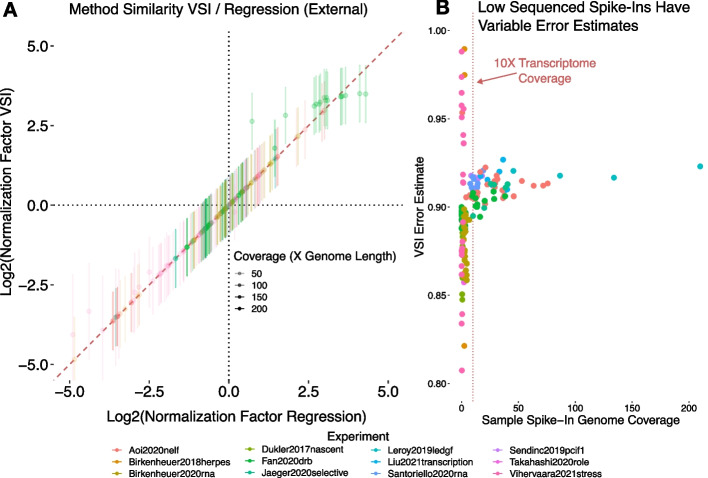
Spike-ins have unusual behavior at the extremes. To assess where our model diverges in behavior from linear regression, we ran the VSI model on data from a number of published experiments [19–32]. Within each experiment, samples were grouped by condition and analyzed within those groups. All samples had Drosophila spike-ins, so annotated Drosophila genes were selected as the invariant set to count over. **A** Comparison of regression factors inferred by linear regression (x-axis) to those inferred by the Bayesian VSI model (y-axis). Estimates are shown along with an error bound of $\pm \sigma$. Notably, the regression estimate (x-axis) and VSI estimate (y-axis) deviate most dramatically when the absolute value of the normalization factor is large. **B** When we plot the depth of coverage of the spike-in (x-axis) against the VSI error estimate (y-axis) shows samples with less than $10\times$ spike-in transcriptome coverage are less consistent than those above this threshold (dotted red line). Of note, error estimates range between 0.8 and 1.0, but when applied to the data they must be converted out of log2 space and multiplied by the normalization factor. Hence the impact of the error will scale with the normalization factor size. In a biological context, this is good—samples with large normalization factors have less confidence indicating poorer experimental efficiency and reproducibility

When running the VSI model on external spike-ins from published data [19–32], we find that it reliably recapitulates the results of naive linear regression (Fig. 2A), but now provides error bars on these estimates. In the regime of small normalization factors (values near zero), both linear regression and the VSI model perform essentially identically. Importantly, when the absolute value of linear regression estimates are large, the VSI approach tends to recover a comparatively lower normalization factor, likely a consequence of the model being more resistant to noise and extreme values than linear regression alone. However, large normalization factors suggest extreme differences in sample efficiencies which should call into question whether the data and spike-in are of sufficient depth and quality to be trusted. A detailed examination of the posterior distribution variance shows higher variability at low spike-in sequencing depth (Fig. 2B). The posterior variance (the variance of the estimated normalization factor after fitting the model) generally improves at depths greater than 10X the dm6 reference transcriptome, using a Drosophila transcriptome length of 30Mb [33]. Unfortunately, the majority of published samples are below this spike-in depth (Additional file 1: Fig. S1). This suggests that most published nascent RNA sequencing experiments using external spike-ins are under-sequenced, which may be a consequence of either an ineffective run-on reaction or a choice to prioritize sample read depth over spike-in read depth.

### Evaluation of error in external and internal normalization

Normalization across invariant regions need not be limited to a spike-in, although an external spike-in is typically preferred. In theory, any set of invariant regions in a sequencing data set that follow a count distribution can be used to estimate a normalization factor between samples. This makes the Virtual Spike-In a versatile and widely useful model for quantifying normalization error across invariant regions.

**Fig. 3:**
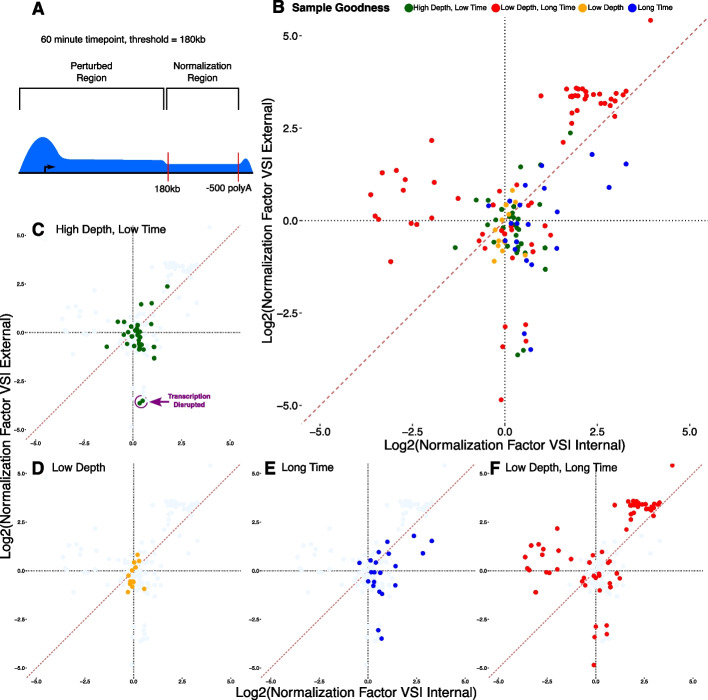
$3^\prime$ Normalization estimates depend on assumed polymerase elongation behavior and sequencing depth **A** A cartoon showing the characteristic shape of nascent RNA sequencing samples after a perturbation. RNA polymerase II loads at the 5$^\prime$ end of genes, thus after a perturbation alterations in transcription levels can only reach a distance that depends on the processivity of RNA polymerase II. In this work we assume 3kb/min and hence for a 60 min experiment the perturbation influences the first 180kb ($60\text {min}\cdot 3\frac{{\text {kb}}}{{\text {min}}}$). **B** We compared external spike-ins (y-axis) to 3$^\prime$ internal normalization across a large collection of previously published data. Samples are colored by whether they **C** meet both time point and depth assumptions (green), **D** have low sequencing depth ($<10$X spike-in transcriptome) (orange), **E** have time points beyond the $3^\prime$ assumed 60 min (blue), or **F** meet neither assumption, being of both low spike-in depth and long time point (red). Notably, two samples in (circled in **C**) meet the coverage and time constraints of the $3^\prime$ normalization approach but involve depletion of NELF under heat shock conditions, which likely alters RNA polymerase elongation

As an example, our model can leverage reads at the $3^\prime$ end of long annotated genes, building on prior work [14, 15] (Fig. 3A). Nascent RNA assays survey engaged RNA polymerases genome-wide, which for any singular time point can be anywhere along the gene. However, in the presence of a perturbation, changes in transcription levels must originate at the 5$^\prime$ end of genes, either by altering RNA polymerase II’s loading and/or release from pausing. Once released, RNA polymerase II then proceeds through the gene at a relatively consistent rate [10, 15]. For example, in human cells RNA polymerase II has an elongation rate of roughly $2-3\frac{{\text {kb}}}{{\text {min}}}$ [34–38], although this rate can be highly variable. Therefore, at short time points, there is insufficient time to alter RNA polymerase II profiles at the 3$^\prime$ ends of a long gene (see Fig. 3A).

Under this model, we note that RNA polymerase II profiles at genes past $\text {Length Threshold}=\text {Elongation Rate}\cdot \text {Time Point}$ should retain a consistent level of baseline transcription unperturbed by the experiment. Using this assumption, the invariant 3$^\prime$ gene regions can be used for normalization between samples. Previous work [14, 15, 39], used a simple linear regression model to determine a normalization factor, defined by the slope of the best fit line, between the two samples using $3^\prime$ regions. However, these models did not establish uncertainty bounds on the accuracy of their normalization factors and did not compare their methodology to external biological spike-ins to quantify its effectiveness.

We leveraged the VSI approach to compare the $3^\prime$ normalization to external spike-in controls (Fig. 3B). For consistency of comparison between different experiments, and considering the typical timelines used, we selected a 180kb ($60\text {min}\cdot 3\frac{{\text {kb}}}{{\text {min}}}$) threshold for all samples when looking at the $3^{\prime }$ invariant region. We also exclude the last 500bps of the annotated gene from our normalization to reduce variance from the characteristic $3^\prime$ bump associated with termination in nascent RNA sequencing experiments. This results in 1198 $3^\prime$ invariant regions used for normalization by the VSI model (roughly 10% of annotated RefSeq genes). Using this set, we found that the correspondence between the $3^\prime$ normalization approach and external spike-ins (Fig. 3B) showed extensive variation. In fact, the internal and external normalization factors were only rarely the same (diagonal line). Thus, we next sought to determine which factors influence the $3^\prime$ normalization method’s fidelity.

We first consider time points below the 60 min threshold utilized. As the posterior estimate of the normalization factor varies dramatically below 10X spike-in coverage (Fig. 2B), we first consider only samples with stable estimates (spike-in coverage $>10$X). For these samples, there is generally good concordance—small differences as most points are near the origin—between the $3^\prime$ normalization and external spike-in approach (Fig. 3C). Notably, two data sets show strikingly lower concordance between the two methods. These two data sets were samples where NELF (negative elongation factor) was depleted and the cells were subjected to heat shock [19]. The lack of concordance between the methods suggests that the depletion of NELF may have had genome-wide effects on RNA polymerase, a condition that calls into question the invariant nature of any internal set.

At low external spike-in depth, inadequate spike-in data may exist for confidence in linear regression. Consistent with this notion, low depth spike-in samples have higher posterior estimate variance (Fig. 2B). However, despite this increased uncertainty, we found good concordance between the spike-in and the $3^\prime$ normalization estimates (Fig. 3D).

Importantly, the $3^\prime$ normalization approach inherently assumes that portions of genes are unreachable at the specified time point of the experiment. By using a uniform 60 min assumption, we could determine whether the concordance between the $3^\prime$ approach and external spike-ins breaks down at longer time points, when the assumed invariant regions can no longer be assured to be unchanged. As expected, when the internal set contains regions that could be varying between the samples (e.g. the time point is longer than the 60 min assumption), there was increasing discordance between the two normalization methods (Fig. 3D,E), particularly when long time points co-occurred with low coverage (Fig. 3F). Intriguingly, even in the data that fail to meet our assumptions (low depth + long time, Fig. 3F) we observe a small cluster of samples close to the origin of the plot. In these scenarios, we achieve concordance between internal and external spike-ins even when all assumptions are violated, as in these cases the perturbation happens to not strongly impact the long gene set used by the VSI normalization.

Collectively, these results suggest that the $3^\prime$ internal normalization approach gives results similar to the linear approximation of external spike-ins when the assumptions of the model are met. This is particularly true when the normalization factors are small (e.g. near the origin in Fig. 3B–F). When the assumptions of the VSI model are violated, either with long time points or disruptions that alter RNA polymerase itself, the two models strongly disagree.

To further characterize this pattern, we next turned our attention to the examination of a single high quality data set that contains multiple time points and roughly average spike-in sequencing depth (GSE96869) [23]. In this study, Dukler et al. treated K562 cells with the natural drug Celastrol, which activates mammalian heat shock response [23]. Cells were then assayed at several time points including 10 min, 20 min, 40 min, 60 min and 160 min. This PRO-seq data set has spike-in sample depth ranging from 0.7 to 1.1X Drosophila transcriptome coverage. Importantly, the cells undergo replicative arrest around the 40 min time point. As before, we employ a 180kb ($60\text {min}\cdot 3\frac{{\text {kb}}}{{\text {min}}}$) threshold for all samples when looking at the $3^{\prime }$ invariant region. For each sample, we compared normalization results using the $3^\prime$ internal normalization to external spike-ins, using both linear regression (VSI) and the ratio based point estimate.

**Fig. 4:**
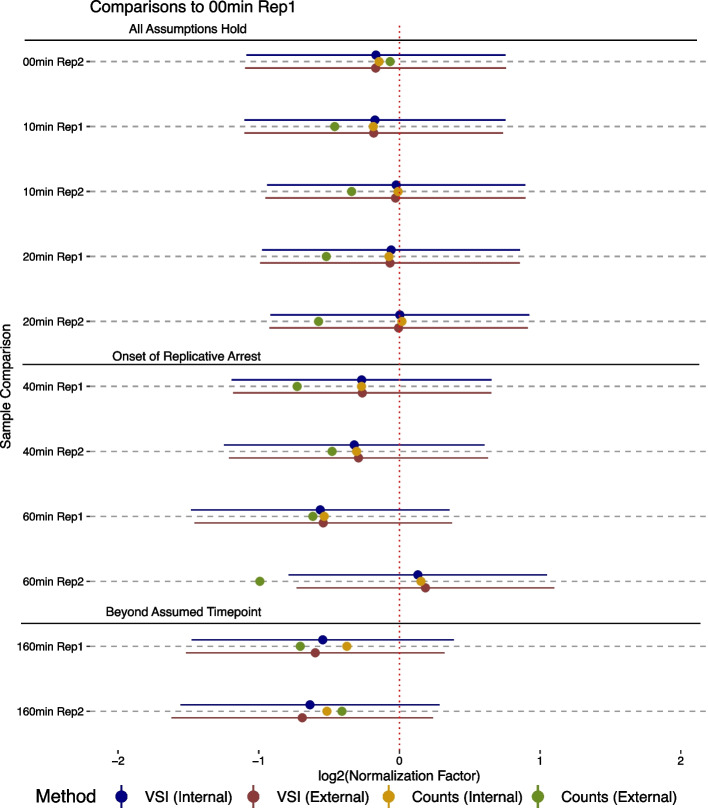
Comparison of all normalization methods on good quality data. We compare normalization factors on a high quality data set [23] (GSE96869) computed by four distinct methods: VSI applied to the internal 3$^\prime$ invariant gene set (blue), VSI applied to an external Drosophila spike-in (red), the ratio approach applied to the 3$^\prime$ invariant gene set (yellow), and the ratio approach applied to the Drosophilia spike-in (green). Error bars are shown for the VSI estimates. The 3$^\prime$ invariant set uses a threshold of 180 kb (60 min), regardless of the data time point. For orientation, we note the normalization factor of zero (red dotted line), the onset of biological replication arrest and the assumed time point for the 3$^\prime$ invariant gene set

We observe that the VSI model shows good concordance between internal ($3^\prime$) and external spike-in estimates of the normalization factor, particularly at early time points (Fig. 4). After the onset of replicative arrest (t=40 min), the internal and external normalization factors begin to diverge, though only modestly in both the 40 min and one of the 60 min time point replicates. As expected, the largest deviations between the $3^\prime$ and external spike-in are observed at 160 min, when the time point is well beyond the 60 min assumed by the internal normalization. At all time points, the single point estimate of the external spike-in deviates substantially from both the linear model estimate of external spike-in and the $3^\prime$ approach, consistent with prior work on normalization approaches [3].

### Downstream effects of normalization

Normalization factors are crucially important in downstream analyses of high throughput sequencing data. To that end, we next compared the results of differential expression analysis on the Dukler data set [23]. For differential expression analysis, we used DESeq2 [2], which uses an internal normalization approach. Specifically, DESeq2 calculates a size factor as the median ratio of counts over every gene in the sample divided by the geometric mean of counts at that gene over all samples. The result is an effective method for normalization that implicitly assumes that most genes are unchanged across the comparison.

**Fig. 5:**
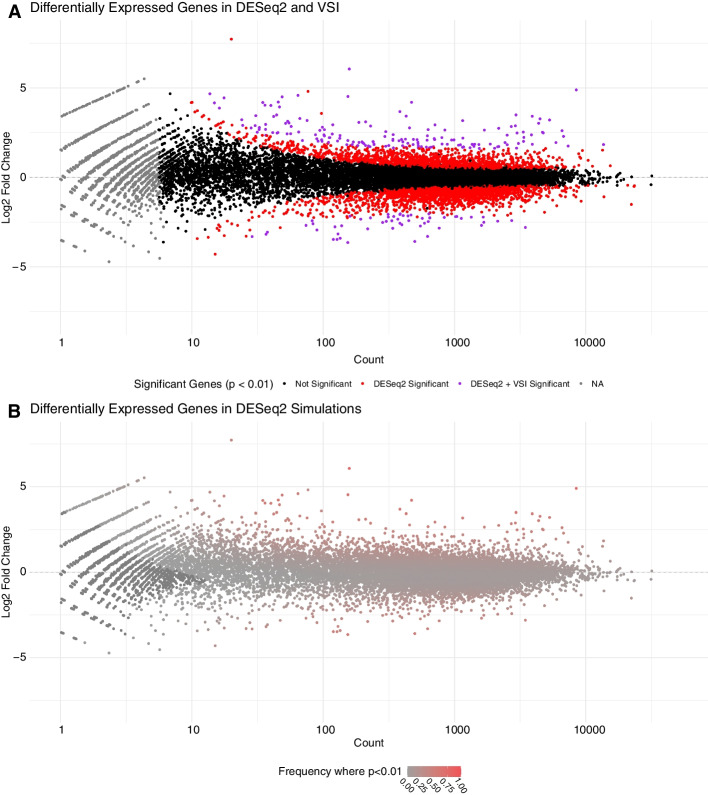
Estimated Normalization Factors Provide Strict Cutoffs for DESeq2 **A** Differential expression analysis by DESeq2 (adj. p-val < 0.01) using size factors estimated from DESeq2 (red) and the VSI model (purple) on $3^\prime$ invariant regions. Note that DESeq2 calls normalization factors “size factors”. The more conservative VSI identified set (purple) is a strict subset of the DESeq2 identified significant set. **B** Consistency of differential expression calls across a broad range of plausible normalization factors. Genes are colored based on the reproducibility of statistically significant differential expression (DESeq2, adj. *p* val $< 0.01$) across 1000 iterations where normalization factors were sampled from the posterior distribution estimated by VSI. Points that appear as significant most often are also those that are called as significant using both DESeq2 size factors and VSI 3$^\prime$ normalization (Panel **A**, purple)

We sought to compare the default DESeq2 size factor approach to the $3^\prime$ internal normalization method. For this comparison, we performed differential expression analysis between the 0 min and 60 min time points (Fig. 5A, 40 min comparison shown in Additional file 1: Fig. S3). We observed that the posterior point estimate for the normalization factor recapitulate a strict subset of genes called as differentially expressed by the automatically estimated size factors (Fig. 5). In simpler terms, it appears that $3^\prime$ internal estimated normalization factors are more conservative, effectively decreasing the set of genes called as significant. Arguably the VSI set is both more conservative and based on a biologically principled invariant set of data compared to the DESeq2 method.

In both cases, a single normalization term is calculated and presumed to be correct. Our earlier comparison to external spike-ins (Fig. 3) suggests two estimators may reach similar but not quite the same normalization factor. Therefore, we next sought to ascertain the extent to which minor, plausible fluctuations in the calculated normalization factor might influence differential expression analysis. To this end, we use a sampling approach. We ran 1000 simulations sampling normalization factors from the posterior distribution estimated by VSI for each of the 4 samples (10 min, 60 min; 2 replicates at each time point). We then ask how often a particular gene is called as significant across the samples. We observe that many of the genes called by DESeq2 as differentially expressed (red dots in Fig. 5A) have relatively low reproduciblity across the range of plausible normalization factors (Fig. 5B) and are therefore potential false positives. Notably, the genes with the highest reproducibility are those found by the VSI $3^\prime$ point estimate (purple dots in Fig. 5A correspond to red dots in Fig. 5B).

## Discussion

We present Virtual Spike-In, a novel approach that uses a hierarchical Bayesian regression model to calculate normalization factors and quantify their uncertainty for nascent transcription datasets. We use this method to compare 3$^\prime$ end normalization in run-on based nascent RNA sequencing experiments to external spike-in controls. We find that while the internal and external normalization rarely perfectly agree, the 3$^\prime$ end normalization shows high concordance to external spike-in controls when assumptions of the method are met. Additionally, normalization is known to have strong effects on analysis results [3], and our work further supports this conclusion (Additional file 2).

While external spike-ins are typically assumed to be the gold standard for normalization of sequencing samples, we find that external spike-ins in published run-on based nascent RNA sequencing experiments are typically under sequenced. Importantly, external spike-ins in nascent RNA sequencing are not the same as those in RNA-seq. This makes the entire normalization process significantly more challenging. Using spike-in nuclei inherently assumes that for every sample, the efficiency of the run-on in the spike-in nuclei closely matches the efficiency of the run-on in the experimental nuclei. There is no reliable mechanism to determine if this assumption is correct. This problem is exacerbated by the relatively low read depth of most external spike-ins in nascent RNA assays. It is critically important that any normalization technique be based on adequate data, as even the best normalization model is limited by the available data.

The alternative to external spike-ins is to use an internal invariant set. Run-on based nascent transcription coupled to a perturbation has a unique invariant set in the $3^\prime$ ends. While 3$^\prime$ end normalization is powerful, it has a number of important limitations compared to an external spike-in. First, the elongation rate of RNA polymerase II in the organism must also be known. At any given elongation rate and time point, a reasonable proportion of genes in the genome must be sufficiently long that invariant regions exist at the time point of interest. While this works well in the human genome, it is likely not the case for organisms with smaller genes and genomes. Even in the human genome, when the normalization factor is estimated on later time points, it is based on increasingly smaller quantities of data, leading to less certainty. With that said, the use of a Bayesian model in this context does make the model robust to a small number of genes to be normalized against. Finally, the 3$^\prime$ end approach cannot be used in the absence of a perturbation or if the perturbation could alter previously loaded RNA polymerase.

In addition to the assumptions made about the model, it is also important to consider the assumptions made about the selected $3^\prime$ regions if performing normalization internally. First and foremost, low expression is a persistent concern across all experiments and must be considered here. Undersequencing is, in general, a problem for normalization (of external spike-ins or of 3$^\prime$ invariant regions) and downstream analysis. Consequently, if a sample is of low sequencing depth, either generally or particularly at the $3^\prime$ end of genes, we recommend it be excluded from further analysis for quality concerns. Likewise, our 3$^\prime$ assessment depends on the accuracy of gene annotations and the presumption that long genes are not in some way atypical. Finally, the presence of intronic bidirectional signals (e.g. same strand overlapping transcription) could be problematic if the bidirectionals both reside within the invariant 3$^\prime$ region and are themselves differentially transcribed. Despite these caveats, one benefit of 3$^\prime$ end normalization is that it can be applied to many previously published run-on based nascent RNA sequencing data sets where an external spike-in is not present.

There are a number of nascent transcription assays that do not use a run-on step, and normalization for these assays present distinct challenges. Metabolic labeling approaches expose live cells to marked nucleotides over some time frame before the experiment [8, 40]. As such, both the profile and signal to noise characteristics of the data are influenced by the time and efficiency of the labeling process. In contrast, mammalian native elongating transcript sequencing (mNET-seq) [41] uses an antibody to pull down a component of the RNA polymerase II complex. As such, normalization of mNET-seq data is conceptually similar to ChIP-seq and should account for antibody efficiency. Further work is needed to characterize both internal and external normalization strategies for metabolic labeling and antibody oriented nascent transcription assays.

The Virtual Spike-In model is versatile. As the input to normalization is counts over a collection of regions, the VSI method can be applied to both internal invariant sets, such as the 3$^\prime$ end normalization used here, and to external spike-in controls. Another notable advantage to the VSI technique is that it establishes error bounds on the calculated normalization factors, an important but often overlooked aspect of the data analysis. Effectively quantifying error in the point estimations of normalization factors is an important addition over the naive linear model. Quantification of error is essential to analyzing nascent RNA sequencing data rigorously. Ultimately, nascent RNA sequencing experiments appear to need a more reliable mechanism for external normalization, which is challenging given the limitations of the underlying protocols.

## Methods

Our model is implemented in the Python programming language using the pymc3 MCMC library [42]. Inference is performed using an adaptive sampler, combining the No-U-Turn Sampler [43] (NUTS) for continuous variables with a Metropolis-Hastings Sampler [44, 45] for discrete variables, using 25,000 iterations after a burn-in period of 2,500 samples. The number of iterations can be increased if a greater assurance of convergence is desired. A larger number of iterations are required for convergence of the discrete distribution due to the use of a Metropolis sampler instead of NUTS (Additional file 1: Fig. S5). Source code is available at https://github.com/Dowell-Lab/virtual_spike_in.

For both the human cell lines and *Drosophila* spike-in, reads were mapped to the hg38 and dm6 reference genomes respectively using the Nascent-Flow pipeline [46]. Counts were determined for all genes using featureCounts [47], considering only the maximally expressed isoform and counting reads per gene including exons and introns.

### Supplementary Information

**Additional file 1**. This file contains supplementary material (additional description of the analysis pipelines) and supplemental figures.

**Additional file 2**. Contains a comma delimited table of the samples and papers that were used for analysis in this study, along with metadata on cell type and whether all analysis steps could be successfully completed on those samples.

## Data Availability

Data for this project was from previously published experiments, with additional metadata available in Additional file 1: Table S1. The datasets analysed in the current study are available in the GEO repository, with the accession numbers GSE144786, GSE143844, GSE106126, GSE130342, GSE96869, GSE141377, GSE139468, GSE117155, GSE150530, GSE104334, GSE128086, GSE122803, GSE121024, GSE127844, and GSE154746.
