# Supplementary material for: Internal and external normalization of nascent RNA sequencing run-on experiments
Source: BMC Bioinformatics. 2024 Jan 12;25:19. doi: 10.1186/s12859-023-05607-3 (PMC10785432; doi:10.1186/s12859-023-05607-3)
Supplement: Supplementary file 1 — Additional file 1. This file contains supplementary material (additional description of the analysis pipelines) and supplemental figures. [file 12859_2023_5607_MOESM1_ESM.pdf]

Internal and External Normalization of Nascent RNA Sequencing Run-On Experiments

Zachary L. Maas and Robin D. Dowell

Supplementary Materials

Analysis of Sequencing Data

All data for this paper was processed using the Nascent-Flow pipeline (, commit e20c72l), a standardized NextFlow pipeline for the analysis of nascent sequencing data. The general flow of this pipeline is as follows:

1. Initial quality control is performed using fastQC
2. Reads are trimmed using BBDuk
3. Post-trimming quality control is done again with fastQC
4. Reads are mapped to the reference genome using HISAT2.
5. Mapped reads are converted into BAM/CRAM files using Samtools.
6. Bedtools is used to generate bedGraph files from the mapped CRAM files.

For input to the VSI NextFlow pipeline developed for this work, we use the BAM and bedGraph files generated using the previous pipeline. Within the VSI pipeline, we take the following analysis steps:

1. Genes within samples are filtered down to a single isoform based on the highest transcribed isoform after length normalization.
2. A single reference isoform list is selected for the entire comparison.
3. The isoform list / ROI list is filtered by length when requested for the analysis (example: the $3'$ methodology)
4. featureCounts is used to count reads over the filtered and processed ROIs for all samples
5. Read counts from each sample are merged into one unified count table
6. The VSI algorithm is run on the merged count table

Characteristics of $3'$ regions

For our $n = 1198$ genes with suitable length for a 180kb threshold, we find that the median length is 288254bp, the mean length is 371005bp, while the minimum length region is 180201bp and the longest region is 2220164bp.

The variance distribution for the VSI is highly skewed

In our testing, we observe that the typical estimated variance for normalization factors is around 1, corresponding to a 2-fold change. However, this estimated variance is noisy, with the 94% Highest Density Interval (HDI) of the variance lying somewhere between 0.10 and 2 in $\log_2$ transformed space. This means the most likely variance on our normalization estimate is somewhere between 1.1-fold and 4-fold, which is a wide range. Because the estimated variance has a skewed distribution, our variance estimates are shifted to be relatively large because of the long tail in our data.

Analysis of Samples

For the sake of consistency of analysis, samples were only analyzed if they could be processed by our analysis pipeline without error or modification to the pipeline code. A full list of files that we used as input for our pipeline and if/why they were excluded can be found in Supplemental File 2. Samples were processed separately in groups of single-end and paired-end samples using the Nascent-Flow pipeline, then analyzed downstream. Once processed, samples were then grouped by experiment and analyzed using the VSI Nextflow pipeline. Two different analyses were performed — one on RefSeq annotated genes in the dm6 genome and one on RefSeq annotated genes constrained to a 180kb / 60 minute $3'$ threshold. 60 minutes was selected among all experiments for the sake of consistency as well as to test the assumptions made in the choice of the $3'$ invariant region. A summary of the data sets used for this study can be found in Table S1.

Data used for this

MCMC convergence and autocorrelation

As discussed in the main text (See Figure S5), the convergence of model parameters is dependent on the sampler used. The NUTS sampler used for the continuous variables is more efficient in exploring the sampling space than Metropolis-Hastings, which is only used to sample from the two discrete

Experiment	*Cell Type*	*SRP Project*	*GSE Accession Number*
Aoi 2020[1]	DLD-1	SRP247346	GSE144786
Barbieri 2020[2]	THP-1	SRP242477	GSE143844
Barbieri 2020	HeLa	SRP242477	GSE143844
Barbieri 2020	iPSC	SRP242477	GSE143844
Birkenheuer 2018[3]	HEp2	SRP121447	GSE106126
Birkenheuer 2020[4]	HEp2	SRP193891	GSE130342
Dukler 2017[5]	K562	SRP102240	GSE96869
Fan 2020[6]	MV4-11	SRP234556	GSE141377
Jaeger 2020[7]	KBM7	SRP227189	GSE139468
Leroy 2019[8]	Myoblast	SRP153901	GSE117155
Liu 2021[9]	CD34+ Erythoblast	SRP261462	GSE150530
Rao 2017[10]	HCT116	SRP124968	GSE104334
Santoriello 2020[11]	A375	SRP188036	GSE128086
Sendinc 2019[12]	MEL624	SRP170033	GSE122803
Sendinc 2019	MEL624	SRP170034	GSE122803
Sendinc 2019	MEL624	SRP170035	GSE122803
Sendinc 2019	MEL624	SRP170036	GSE122803
Sendinc 2019	MEL624	SRP170037	GSE122803
Sendinc 2019	MEL624	SRP170038	GSE122803
Sendinc 2019	MEL624	SRP170039	GSE122803
Sendinc 2019	MEL624	SRP170040	GSE122803
Sendinc 2019	MEL624	SRP170041	GSE122803
Takahashi 2020[13]	HEK293T	SRP164752	GSE121024
Vihervaara 2021[14]	K562	SRP187541	GSE127844
Vihervaara 2021	K562	SRP187541	GSE154746

Table S1: Accession Numbers for Analyzed Projects

variables. Convergence of the discrete distributions is the limiting step in model convergence.

References

[1] Aoi, Y., Smith, E.R., Shah, A.P., Rendleman, E.J., Marshall, S.A., Woodfin, A.R., Chen, F.X., Shiekhattar, R., Shilatifard, A.: NELF Regulates a Promoter-Proximal Step Distinct from RNA Pol II Pause-Release. Molecular Cell **78**(2), 261–2745 (2020). doi:10.1016/j.molcel.2020.02.014

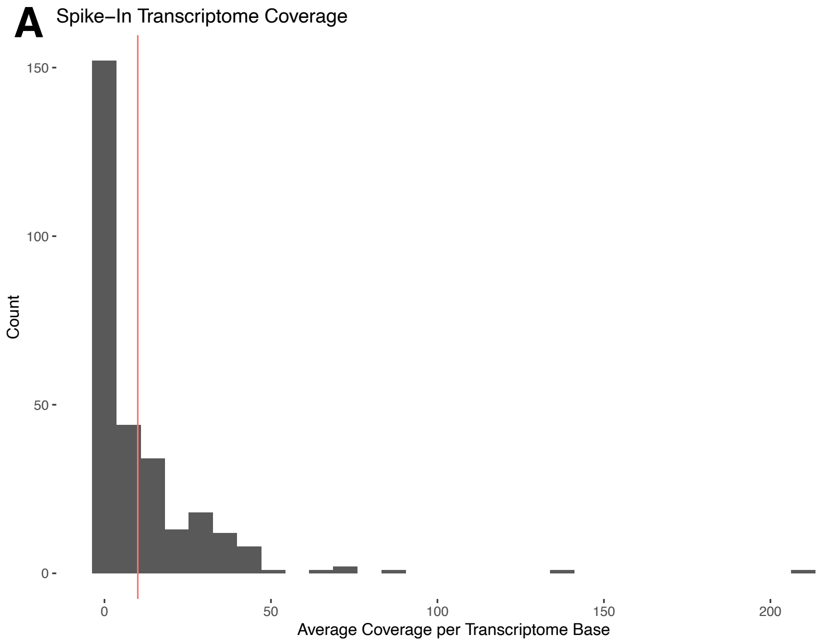

Figure S1: **Depth of spike-in sequencing across data sets from the literature.** Red vertical line is at 10X transcriptome coverage of Drosophilia. Most data sets are under-sequenced relative to a minimum threshold of 10x coverage for use in normalization.

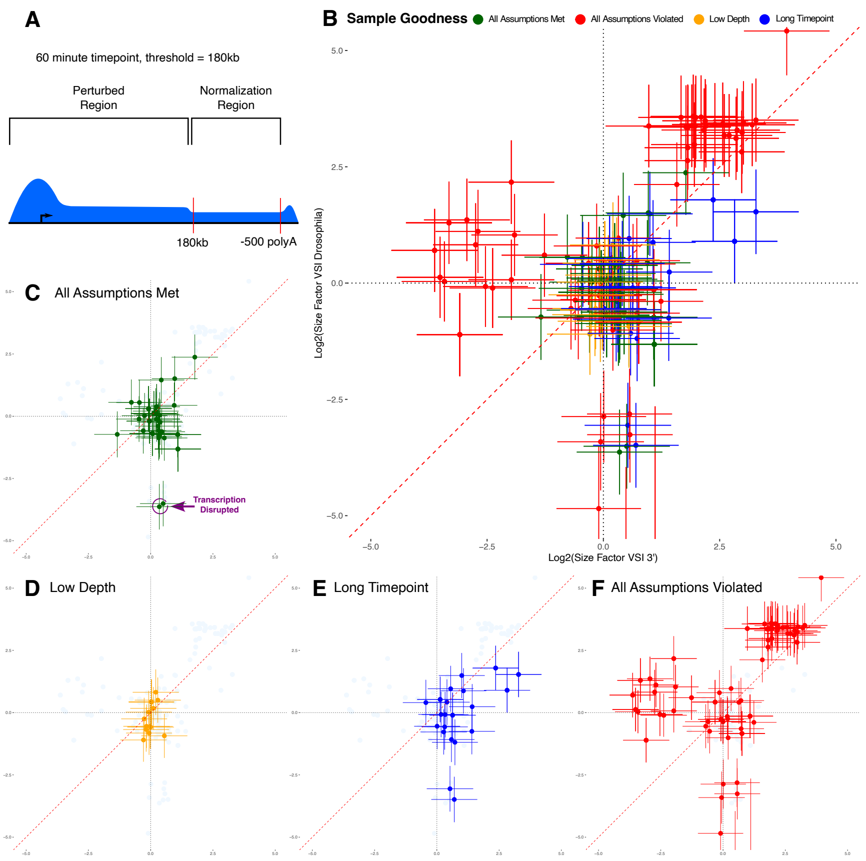

Figure S2: **Comparison of internal 3′ normalization to exogenous spike-ins**. This figure is the same as Figure 3 but each panel B-F now with error bars.

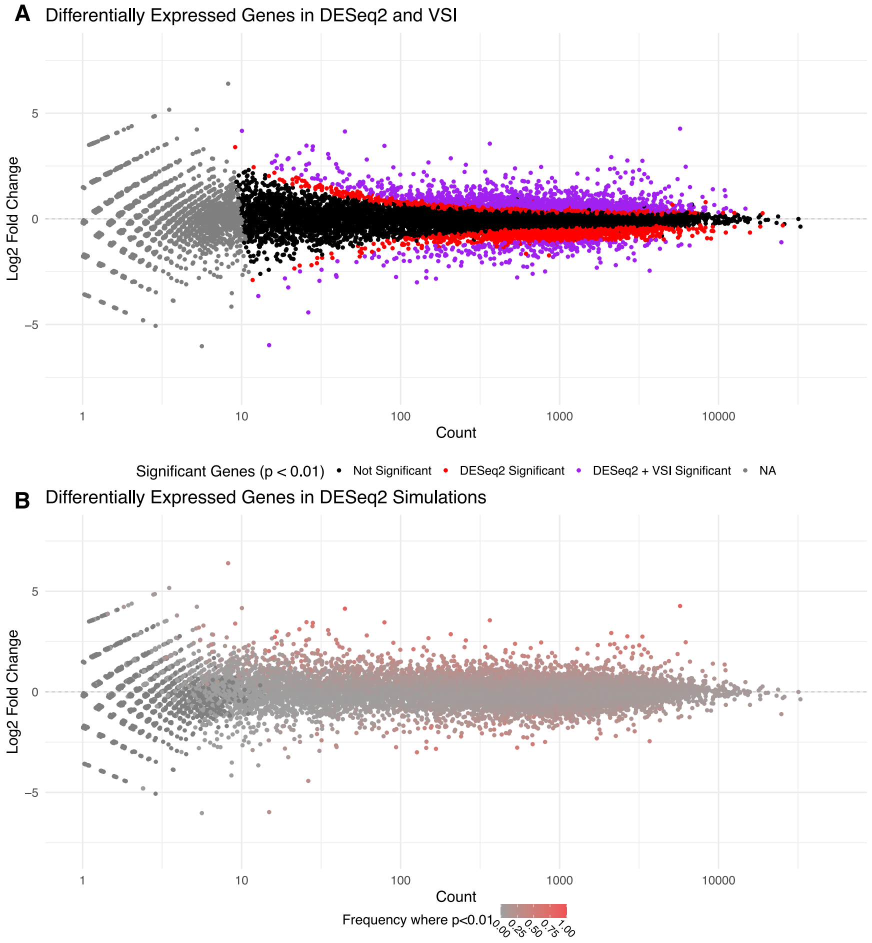

Figure S3: The same as Figure 5, but using the 40 minute for comparison instead of the 60 minute timepoint.

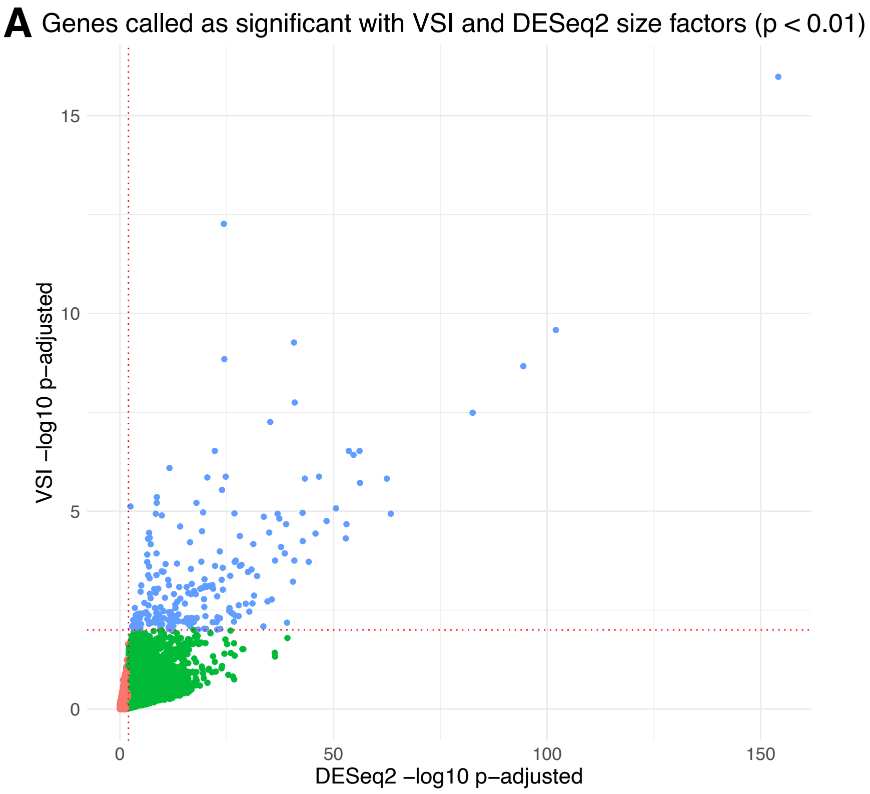

Figure S4: **Comparison of DESeq2 to VSI normalization.** In the DESeq2 results shown in Figure 5A, using size factors estimated from the VSI 3′ internal approach instead of those naively estimated by DESeq2. Notably, for this data set, no stricter p-value cutoff on DEseq2 results (x-axis) would produce the same gene set as called by the VSI method (y-axis), yet VSI is a strict subset of the DESeq2 calls.

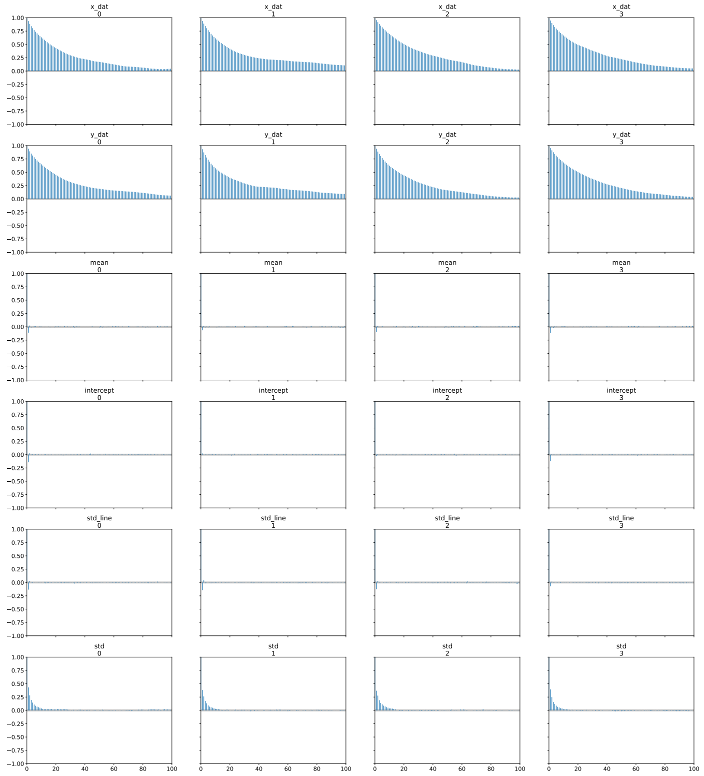

Figure S5: Parameter autocorrelation across 4 runs (chains) (columns, left to right) for a single pairwise comparison (SRR5364303 vs SRR5364304) corresponding to the Dukler 2017[5] 0 minute replicates 1 and 2. Estimates of the negative binomial distributions (top half of plot) show autocorrelation for much longer than estimates of the mean and variance (bottom half of plot). Some degree of autocorrelation is expected when fitting count data under the assumption that the $3'$ region is invariant, as the choice of an invariant region implies that values should be correlated across samples. Additionally, some degree of autocorrelation is likely unavoidable, because in count data both autocorrelation and overdispersion can have the same causes[15]

[2] Barbieri, E., Hill, C., Quesnel-Vallières, M., Zucco, A.J., Barash, Y., Gardini, A.: Rapid and Scalable Profiling of Nascent RNA with fastGRO. Cell Reports **33**(6), 108373 (2020). doi:10.1016/j.celrep.2020.108373

[3] Birkenheuer, C.H., Danko, C.G., Baines, J.D.: Herpes Simplex Virus 1 Dramatically Alters Loading and Positioning of RNA Polymerase II on Host Genes Early in Infection. Journal of Virology **92**(8), 02184–17 (2018). doi:10.1128/JVI.02184-17

[4] Birkenheuer, C.H., Baines, J.D.: RNA Polymerase II Promoter-Proximal Pausing and Release to Elongation Are Key Steps Regulating Herpes Simplex Virus 1 Transcription. Journal of Virology **94**(5), 02035–19 (2020). doi:10.1128/JVI.02035-19

[5] Dukler, N., Booth, G.T., Huang, Y.-F., Tippens, N., Waters, C.T., Danko, C.G., Lis, J.T., Siepel, A.: Nascent RNA sequencing reveals a dynamic global transcriptional response at genes and enhancers to the natural medicinal compound celastrol. Genome Research (2017). doi:10.1101/gr.222935.117

[6] Fan, Z., Devlin, J.R., Hogg, S.J., Doyle, M.A., Harrison, P.F., Todorovski, I., Cluse, L.A., Knight, D.A., Sandow, J.J., Gregory, G., Fox, A., Beilharz, T.H., Kwiatkowski, N., Scott, N.E., Vidakovic, A.T., Kelly, G.P., Svejstrup, J.Q., Geyer, M., Gray, N.S., Vervoort, S.J., Johnstone, R.W.: CDK13 cooperates with CDK12 to control global RNA polymerase II processivity. Science Advances **6**(18), 5041 (2020). doi:10.1126/sciadv.aaz5041

[7] Jaeger, M.G., Schwalb, B., Mackowiak, S.D., Velychko, T., Hanzl, A., Imrichova, H., Brand, M., Agerer, B., Chorn, S., Nabet, B., Ferguson, F.M., Müller, A.C., Bergthaler, A., Gray, N.S., Bradner, J.E., Bock, C., Hnisz, D., Cramer, P., Winter, G.E.: Selective Mediator dependence of cell-type-specifying transcription. Nature Genetics **52**(7), 719–727 (2020). doi:10.1038/s41588-020-0635-0

[8] LeRoy, G., Oksuz, O., Descostes, N., Aoi, Y., Ganai, R.A., Kara, H.O., Yu, J.-R., Lee, C.-H., Stafford, J., Shilatifard, A., Reinberg, D.: LEDGF and HDGF2 relieve the nucleosome-induced barrier to transcription in differentiated cells. Science Advances **5**(10), 3068 (2019). doi:10.1126/sciadv.aay3068

[9] Liu, N., Xu, S., Yao, Q., Zhu, Q., Kai, Y., Hsu, J.Y., Sakon, P., Pinello, L., Yuan, G.-C., Bauer, D.E., Orkin, S.H.: Author Correction: Transcription factor competition at the γ-globin promoters controls hemoglobin switching. Nature Genetics **53**(4), 586–586 (2021). doi:10.1038/s41588-021-00834-x

[10] Rao, S.S.P., Huang, S.-C., Glenn St Hilaire, B., Engreitz, J.M., Perez, E.M., Kieffer-Kwon, K.-R., Sanborn, A.L., Johnstone, S.E., Bascom, G.D., Bochkov, I.D., Huang, X., Shamim, M.S., Shin, J., Turner, D., Ye, Z., Omer, A.D., Robinson, J.T., Schlick, T., Bernstein, B.E., Casellas, R., Lander, E.S., Aiden, E.L.: Cohesin Loss Eliminates All Loop Domains. Cell **171**(2), 305–32024 (2017). doi:10.1016/j.cell.2017.09.026

[11] Santoriello, C., Sporrij, A., Yang, S., Flynn, R.A., Henriques, T., Dorjsuren, B., Custo Greig, E., McCall, W., Stanhope, M.E., Fazio, M., Superdock, M., Lichtig, A., Adatto, I., Abraham, B.J., Kalocsay, M., Jurynec, M., Zhou, Y., Adelman, K., Calo, E., Zon, L.I.: RNA helicase DDX21 mediates nucleotide stress responses in neural crest and melanoma cells. Nature Cell Biology **22**(4), 372–379 (2020). doi:10.1038/s41556-020-0493-0

[12] Sendinc, E., Valle-Garcia, D., Dhall, A., Chen, H., Henriques, T., Navarrete-Perea, J., Sheng, W., Gygi, S.P., Adelman, K., Shi, Y.: PCIF1 Catalyzes m6Am mRNA Methylation to Regulate Gene Expression. Molecular Cell **75**(3), 620–6309 (2019). doi:10.1016/j.molcel.2019.05.030

[13] Takahashi, H., Ranjan, A., Chen, S., Suzuki, H., Shibata, M., Hirose, T., Hirose, H., Sasaki, K., Abe, R., Chen, K., He, Y., Zhang, Y., Takigawa, I., Tsukiyama, T., Watanabe, M., Fujii, S., Iida, M., Yamamoto, J., Yamaguchi, Y., Suzuki, Y., Matsumoto, M., Nakayama, K.I., Washburn, M.P., Saraf, A., Florens, L., Sato, S., Tomomori-Sato, C., Conaway, R.C., Conaway, J.W., Hatakeyama, S.: The role of Mediator and Little Elongation Complex in transcription termination. Nature Communications **11**(1), 1063 (2020). doi:10.1038/s41467-020-14849-1

[14] Vihervaara, A., Mahat, D.B., Himanen, S.V., Blom, M.A.H., Lis, J.T., Sistonen, L.: Stress-induced transcriptional memory accelerates promoter-proximal pause release and decelerates termination over mitotic divisions. Molecular Cell **81**(8), 1715–17316 (2021). doi:10.1016/j.molcel.2021.03.007

[15] Barron, D.N.: The Analysis of Count Data: Overdispersion and Autocorrelation. Sociological Methodology **22**, 179–220 (1992). doi:10.2307/270996
